# Caretaker-adolescent communication on sexual and reproductive health: a cross-sectional study in Unguja-Tanzania Zanzibar

**DOI:** 10.1186/s12889-017-4591-2

**Published:** 2017-07-18

**Authors:** Saada A. Seif, Thecla W. kohi, Candida S. Moshiro

**Affiliations:** 1 Department of Nursing Management, School of Nursing, Muhimbili University of Health and Allied Sciences, P.O. Box 65001, Dar es Salaam, Tanzania; 2 Department of Nursing Management, School of Nursing, Muhimbili University of Health and Allied Sciences, P. O. Box, 65001 Dar es Salaam, Tanzania; 3 Department of Epidemiology and Biostatistics, School of Public health and Social Sciences, Muhimbili University of Health and Allied Sciences, P. O. Box, 65001 Dar es Salaam, Tanzania

**Keywords:** Adolescents, Caretakers, Sexual and reproductive health, Communication, Parents, Sexuality

## Abstract

**Background:**

Caretakers/parents/caregivers/guardians play important roles in improving Sexual and Reproductive Health (SRH) of adolescents. Caretaker-adolescent sexual communication suggested to influence young people’s sexual behaviours. Despite this significance, the communication is believed to be low in Unguja due to the increase of risky sexual behaviours among adolescents. This study assessed the pattern of such communication using IMB model as a framework.

**Methods:**

This is a cross-sectional study targeted caretakers of adolescents aged 15–19. One thousand caretakers of adolescents were interviewed using structured questionnaire. Comparison between male and female caretakers on discussing different SRH topics to both sexes of adolescents was made. The mean-score difference of overall communication was examined using Univariate analysis of variance (ANOVA). Bivariate correlation and simple path analysis via regression was conducted to determine the association of IMB variables in relation to communication practice.

**Results:**

This study finds 40.7% of caretakers had ever communicated with their adolescents on SRH matters and 9.2% reported to have had communicated in the past 30 days. The weighted topic measure revealed only 26.5% of caretakers communicated with their adolescents. Both caretakers communicated more with their female adolescents. The communication was more common between same sex and between caretakers and their biological adolescents (*p* < 0.000). Both male and female caretakers mostly discussed sexual abstinence to female adolescents while to male adolescents, HIV/STIs was mostly discussed by female caretakers and pregnancy by male caretakers. The least discussed topics to both sexes are safer sex and other contraceptives use. The bivariate correlations suggested that IMB constructs were inter-related and associated with communication practice.

**Conclusion:**

Caretakers-adolescents communication on SRH in Unguja is low and it is not comprehensive. Caretakers fail to communicate with their adolescents on sensitive issues but do so on less sensitive ones. The pattern of communication found to vary across gender of caretaker and that of adolescent and depends on the nature of relationship between caretaker and adolescent. There is gender differences in selecting SRH topics of discussion. Interventions programmes have to include strategies that enhance caretaker’s information, motivation and skills so as to improve SRH communication between caretakers and adolescent.

## Background

Risky sexual behaviours acquired during adolescence, such as early sexual initiation, unprotected intercourse, and multiple sexual partners, can place young people at risk of HIV infections and sexually transmitted infections (STIs), teenage pregnancy and abortion complications. In Unguja, it was reported that adolescents like to engage in uncontrolled leisure activities, entertainment, music, alcohol, and sexual intercourse at a young age and with different people [1]. By the age of 18 years, 20% of the population in Unguja reported to have had practiced sexual intercourse, and the HIV reports show that about 114 (43%) out of 265 of people who have been infected are young people of age 15 and 24 years [2, 3] (Although these young ones could have been infected from birth, they may be a source of HIV transmission if they get involved in unprotected sex). In Zanzibar, 6% of women In Zanzibar, 6% of women aged 15-19 years have commenced childbearing [4]. Concerns about these high rates prompted to ask questions about what and how caretakers and their children talk about sexual health.

Parents play a substantial role in the gender development and sexual socialization of their children [5]. Parent-child communication is one of the parental influence that has received a great deal of attention due to its relationship to adolescent’s sexual risk-taking [6]. When parents/caretakers discuss topics of SRH with their young ones, a range of important psychosocial attributes including knowledge, interpersonal communication skills like sexual negotiation skills, and self-efficacy in condom use are believed to increase [7–9]. Furthermore, adolescents and children often cite their parents as their preferred source of education about sex, and organised prevention and education efforts continue to advocate active parental involvement in children’s sexual socialization [10]. Therefore, caretaker-adolescent communication on SRH to enable adolescents to make informed decision about their sexual health cannot be underestimated.

In Sub-Sahara Africa (SSA), studies on caretaker-adolescent communication on SRH are increasing [11], however; in Tanzania this area has not been well studied. The few studies that are available have focused exclusively on adolescents [12–15], and others are primarily done in rural areas [16] where social structures are different from social structures in urban areas, and have adopted qualitative methodologies [16, 17] which does not provide numerical estimation of the amount of communication, thus limit the generalization of the findings. In Unguja-Zanzibar, to our knowledge, there are only two qualitative studies documented which revealed that caretakers and adolescents have positive perceptions about caretaker-adolescent communication on SRH [1, 18], and one quantitative study done in Urban district which revealed that 54% of caretakers were willing to provide SRH information to their adolescents children [19].

### Theoretical framework: Information-motivation-Behavioural skills (IMB) model

**Fig. 1:**
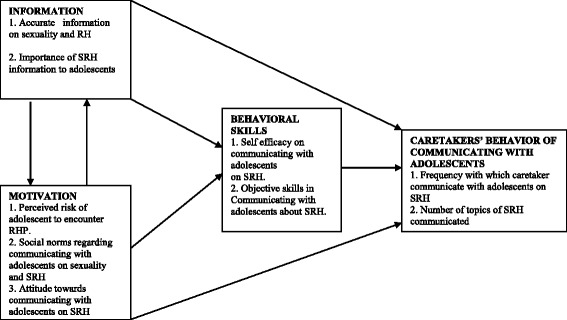
The Information-Motivation-Behavioral Skills (IMB) Model (*Source: Fisher and Fisher,1992)*

Theory-based research is necessary to identify the determinants of SRH communication which can be targeted in intervention. The current study utilized Information-Motivation-Behavioral skills (IMB) model [20] as a framework to guide the SRH communication assessment. This model incorporates elements from other theories such as the Health Belief Model (HBM), and it has been described as being a simple construct to explain complex health behaviours [21]. According to the IMB model (Fig. 1), information (knowledge), motivation and behavioural skills are the fundamental determinants of the initiation and maintenance of health behaviours [20]. This model hypothesizes that if someone is well-informed about the behaviour, and is motivated to perform the behaviour (e.g. by perceiving vulnerability, having less restrictive social support and has positive attitudes towards the behaviour), and has the necessary skills and confidence in their ability to do so across various situations, then this person is more likely to perform a health behaviour [22]. It must be noted that information and motivation are potentially independent constructs which means that well informed individuals are not necessarily motivated to engage in health promotion behaviors or well motivated individuals are not necessarily well informed about health promotion practices. The behavioral skills represent a final common pathway for predicting complex acceptable behaviors [23].

The current study therefore assessed caretaker-adolescent communication on SRH using IMB model as a framework. The identification of communication prevalence and the exploration of a relationship of IMB constructs to communication practice will provide pertinent information for planning appropriate intervention programmes for parents to prevent and reduce risky sexual behaviours among their adolescents.

## Methods

### Setting and study population

This study was conducted in Unguja-Zanzibar, and it involved all the 3 regions of Unguja and all the 6 districts contained within the regions. The study population were all caretakers of adolescents aged 15–19 years. As for this study a caretaker means a parent, caregiver or guardian of adolescent. This definition was conceived because in Tanzanian context, many are extended families and that it is common adolescent live under the care of his/her parents, aunt, uncle, sister/brother or grandparents. According to the *National Population and Housing Census of 2012*, there were about 302,293 people aged between 35 and 80 years; among them, 147,470 were male and 154,823 were female [24]. Both male and female caretakers were included in the study. Study subjects were eligible to participate in the study if caretaker provide voluntary consent, being either the biological parent or a parent figure who must have stayed continuously with the adolescent for at least 2 years prior to the survey. Caretakers who stayed with young people who are married were considered ineligible in the study.

### Design and sample size

We conducted a cross-sectional community-based household survey among caretakers of adolescents of 15 to 19 years of age to assess their communication practice on SRH with their adolescents. IMB model was used as a framework to guide the assessment process. A total of 1000 from the sampled individuals participated in the study with 206 (20.6%) men and 794 (79.4%) women. Twenty respondents refused to participate due to their personal reasons thus yielding a response rate of 97%.

### Sampling procedure

A three-stage probability sampling technique was used to select the individuals. Simple random sampling was used to select 12 wards and 36 shehias. A shehia is the lowest administrative authority in the hierarchy of regional administration [25]. Systematic random sampling was then used to select 28 households from a sampling frame consisting of approximately 450 houses [26]. After the first household, the next 16th household with eligible subjects was chosen for interview. This process continued until the target sample size was obtained. In each household, the parent or caretaker who was defined as the person with the primary responsibility for providing supervision and care for the adolescent was interviewed. If both male and female caretakers were present, a male caretaker was deliberately chosen because of the prior experience that male caretakers are difficult to reach due to the nature of their work compared to female caretakers. In houses with multiple households (for example compound houses), one household was randomly selected for interviews.

### Data collection

Data were collected from March to April 2014 using face-to-face interviews with structured questionnaire containing closed-ended questions (Table 1). A standardized scale has not yet been developed to measure the components of the IMB model for caretaker-adolescent’s SRH communication behavior. An IMB measure used in another study [27] on behavioral change was used in the present study and guided the development of information, motivation and behavioral skills scale to measure SRH communication practice. Content validity of this scale was assessed through peer review and internal consistency reliability for each measure was calculated through a pilot study on 50 caretakers. After revisions, the final version of the scale was prepared. It took 10 min to complete the questionnaire and it was administered by trained interviewers in Kiswahili language spoken by all participants. Interviews were conducted in privacy to avoid eavesdropping, and to ensure openness and truthful responses.

**Table 1:** Summary of data collection tool

S/N	Objective	Variables	Type of scale (range)	No. of Items	Eg. of item to construct the tool
1	To determine Caretakers’ information (knowledge) on adolescent’s SRH and its importance in Unguja-Zanzibar.	knowledge	Binary (0–15)	2, with 15 sub- items	What topics about adolescents’ sexual health need to be communicated to adolescents?
2	To determine Caretakers’ motivation (perceived risk, attitude and social norms) towards communication with adolescents about SRH in Unguja-Zanzibar.	(i) perceived risk,	Likert (1–12)	3	Please grade the risk of your adolescents male to get STIs/HIV
		(ii) attitude,	Likert (1–20)	5	Communicating with adolescent about SRH will promote promiscuity
		(iii) social norms	Likert (1–12)	3	Most of my relatives/friends talks to their adolescents about SRH
3	To determine Caretakers’ behavioral skills (self efficacy and perceived skills) to communicate with adolescents about SRH in Unguja-Zanzibar	i) self efficacy	Likert (1–16)	4	I can describe the act of talking to my Adolescent about SRH as:
		(ii)perceived Skills	Likert (1–16)	4	I can describe my ability to talk to my adolescent about SRH as
4	To determine caretaker-adolescent sexuality communication (frequency and contents of communication) in Unguja-Zanzibar	Frequency and Contents of communication	Likert (1–28)	9	How frequently do you talk to your adolescent male/female ab out HIV and AIDS and STIs?

### Measures

### Demographic characteristics

General demographic information was measured by seven items which are age, sex, marital status, level of education, occupation, number and sex of adolescents one has, and relationship a caretaker has with the adolescent.

#### IMB constructs

- **Information**: This was measured with 15 items to assess knowledge on the contents of SRH and importance of SRH information to adolescent’s life. The contents are divided into biological aspects (e.g. Menstruation), preventive aspects (e.g. safer sex), and associated risk aspects (e.g. HIV and pregnancy). Participants were required to mention spontaneously the contents and importance of SRH. (e.g. what topics of SRH a caretaker has to discuss with the adolescent? What is the importance of SRH information to adolescents life?) One point was awarded for a correct-match mentioned item. The maximum score of the information construct is 15 points, (Cronbach’s Alpha coefficient was 0.93).
- **Motivation:** This was measured with 11 items on ***(i)Caretakers perceived risk:*** Three Likert type items were used to assess the caretaker’s perceived risk of his/her adolescents to get reproductive health problems. The question was: Grade the risk of your adolescent female to get (i. pregnancy, ii. HIV/STIs, iii. to do abortion) or of your adolescent male to impregnate a girl or to encourage abortion. The score ranged from 1 = no risk at all to 4 = high risk. The maximum score is 12 points in which a high score indicates more feeling of vulnerability for his/her adolescent to experience reproductive health problems (Cronbach’s Alpha coefficient was 0.81). ***(ii)Caretaker’s social norms***: Three Likert type items were used to assess caretaker’s perceptions of social support from significant others in communicating about SRH matters to adolescents (e.g. family, friends and relatives). Example of the question was: My friends and relatives talk to their adolescents about SRH matters. The score ranged from 1 = strongly disagree to 4 = strongly agree. The maximum score is 12 points and a high score indicates less restrictive norms in communicating SRH matters with adolescents. Item number 3 was reversed so that higher score meant less restrictive social norms (Cronbach’s Alpha coefficient was 0.41). ***(iii)Caretaker’s attitudes:*** Five Likert type items were used to assess caretaker’s attitude towards communicating with adolescents on SRH matters. Example of the question was; Communicating SRH matters with adolescents will promote promiscuity. The score ranged from 1 = strongly disagree to 4 = strongly agree. The maximum score was 20, and all items were reversed so that higher score means positive attitude (Cronbach’s Alpha coefficient was 0.63). The total motivation score was 44 points.
- **Behavioral skills:** Behavioral skills in communicating with adolescents about SRH were measured on: ***(i)Caretaker’s perceived self efficacy:*** Four Likert scale type items were used to measure perceived easiness in communicating SRH matters with adolescents. Example of the question was: As a female caretaker, I can describe the action of talking to my male adolescent as: The score ranged from 1 = very hard to 4 = very easy, and the maximum score was 16 points (Cronbach’s Alpha coefficient was 0.71). ***(ii)Caretaker’s perceived Objective skills***. Four Likert scale type items were used to measure perceived effectiveness of communicating SRH matters with adolescents. Example of the question was; I can describe my ability to talk to my adolescent as: The score ranged from 1 = very ineffective to 4 = very effective, and the maximum score was 16 points. The total behavioral score was 32 points (Cronbach’s Alpha 0.78).

#### **Communication practice**

Caretakers-adolescents communication on SRH was assessed using two measures; global communication measure and the detailed examination of communication of specific sexual topics (overall measure of communication). These procedures were used to facilitate the objective and subjective assessment of parent–child understanding of sexual communication.

- **Global measure of caretakers-adolescents SRH communication**
  We used this measure to assess whether caretakers had ever communicated with their adolescents on SRH matters. Two questions on likert scale asked how frequently caretakers had communicated with their adolescents (either biological child, child of other family member or both) on SRH issues, and how frequently they had communicated in the past 30 days. The response options was 1 = never to 4 = a lot. For the purpose of this analysis, never and hardly ever were recoded as 0 = never, and sometimes and a lot as 1 = ever in both questions.
- **Overall measure of caretakers-adolescents communication on SRH**
  The overall communication was measured using a weighted topics measure of family sexual communication scale which was developed by Fisher, (1987) [27]. The scale combines a relatively objective measure (the number of topics discussed out of 9 topics) with a more subjective one (the extent of discussion). In the present study, the instrument asks respondents to indicate on a Likert the extent to which seven specific sexual topics have been discussed to either female or male adolescents, (abstinence, pregnancy, safer sex, HIV/STIs, contraceptives use, abortion, and homosexuality). Example of the question was: How frequently do you talk to your adolescent male/female about pregnancy? The response options was 1= never to 4 = a lot. Scores are computed by summing all items, with higher scores indicating greater amounts of sexual communication between parents and adolescent. The maximum score is 28 point, (Cronbach’s alpha 0.81). For the purpose of this analysis caretakers will also be regarded as they communicate if they report communication of at least four topics at the frequency of sometimes or a lot.

### Data analysis

Data were entered and analysed using Statistical Package for Social Sciences (SPSS) version 22. Descriptive statistics were used, frequency distribution and cross-tabulation used to summarize socio-demographic data and the SRH topics discussed to either sex of adolescents by male and female caretakers, and the difference between them was examined using Pearson’s Chi-square (*χ* ^2^). The mean score-sex difference of all IMB constructs was examined using a t-test. The overall communication prevalence was computed and the mean difference was compared between different socio-demographic variables using Univariate analysis of variance (ANOVA). Bivariate correlation between IMB variables and communication practice was calculated, and simple path analysis via regression was conducted to determine the association of IMB variables in relation to communication practice. Pearson correlation and regression coefficient was reported and the cut-off point for level of significance was set at a two-sided, *p*-value <0.05.

## Results

### **Characteristics of respondents**

A total of 1000 participants, 205 (20.5%) men and 795 (79.5%) women were interviewed. The age of the respondents ranged from 35 to 80 years with a mean = 45.6 and standard deviation of 10.8. Comparing to male caretakers, significantly more female caretakers were in the age group 35–39 years, and more (77.5% vs. 71.7%) live with their biological adolescents. Male caretakers significantly differed from female caretakers on marital status and occupation as majority of male 193 (94.1%) were married, and 131 (63.9%) were either farmer or fisherman. There were no other significant differences between men and women on other demographic characteristics (Table 2).

**Table 2:** Sex difference of socio-demographic characteristics of caretakers

Characteristics	Male (n = 205)	Female (n = 795)	p-value ^a^
	Number (%)	Number (%)	
Age (years)			
35–39	48 (23.40)	343 (43.1)	<0.001
40–49	44 (21.50)	217 (27.3)	
50–59	53 (25.9)	164 (20.6)	
60+	60 (29.3)	71 (8.9)	
Marital status			
Single	4 (2.0)	12 (1.5)	0.01
Married	193 (94.1)	698 (87.8)	
Divorced	4 (2.0)	16 (2.0)	
Widower / Widow	4 (2.0)	69 (8.7)	
Education level			
No formal education	55 (26.8)	182 (22.9)	0.44
Primary education	65 (31.7)	280 (35.2)	
Secondary and higher education	85 (41.5)	333 (41.9)	
Occupation			
Farmer/fisherman	131 (63.9)	400 (50.3)	<0.001
Employed	29 (14.1)	59 (7.4)	
Petty business	43 (21.0)	190 (23.9)	
Housewife/housemother/father	2 (1.0)	146 (18.4)	
Caretaker’s adolescents’ sex			
Caretakers having male adolescents only	49 (23.9)	217 (27.3)	0.34
Caretakers having female adolescents only	82 (40.0)	332 (41.8)	
Caretakers having both male and female adolescents	74 (36.1)	246 (30.9)	
Relationship with adolescent			
Biological child	147 (71.7)	616 (77.5)	0.05
Child of other family member	14 (6.8)	27 (3.4)	
Both biological and of other family member	44 (21.5)	152 (19.1)	

^a^Chi square test

### Prevalence of caretakers-adolescents communication on SRH (global measure)

The results showed that 407 (40.7%) caretakers had ever communicated with their adolescents on SRH issues and 92 (9.2%) caretakers reported to have had communicated with their adolescents in the past 30 days. Female caretakers, caretakers who stayed with their biological adolescents, those of 50–59 years of age, and those having female adolescents were significantly more likely to have had communicated (ever and in the past 30 days) with their adolescents than their counterparts (Table 3).

**Table 3:** Communication on SRH (ever and in the past 30 days) among caretakers of adolescents (*N* = 1000)

Characteristic	Total	Ever communicate (n = 407) Number (%)	*p*-value ^a^	Communicate in the past 30 days (n = 92) Number (%)	*p- value* ^a^
Sex					
Female	795	344 (43.3)	0.01	79 (9.9)	0.01
Male	205	63 (30.7)		13 (6.3)	
Age groups (years)					
35–39	391	141 (36.1)	0.04	33 (8.4)	0.83
40–49	261	108 (41.4)		26 (10.0)	
50–59	217	104 (47.9)		19 (8.8)	
60+	131	54 (41.2)		14 (10.7)	
Relationship with adolescent					
Biological adolescent	763	326 (42.7)	<0.001	82 (10.7)	0.01
Non biological adolescent	41	15 (36.6)		2 (4.9)	
Have both biological and non biological adolescent	196	66 (33.7)		8 (4.1)	
Caretaker’s adolescents’ sex					
Caretakers having male adolescents only	266	51 (19.2)	<0.001	11 (4.1)	0.001
Caretakers having female adolescents only	414	182 (44.0)		53 (12.8)	
Caretakers having both male and female adolescents	320	174 (54.4)		28 (8.8)	

^a^Chi square test

### Overall measure of SRH communication

The prevalence of communication by looking on the number of topics a caretaker reported to communicate at the frequency of sometimes and or a lot, only 265 (26.5%) of caretakers communicated at least four topics of SRH and thus were termed as they communicate. On the other hand, assessment of SRH communication using the weighted topics measure of family sexual communication scale revealed that the participant’s score ranged from 4 to 25 points (maximum 28 pints) with an average of 10.28 and standard deviation of 4.76.

This study finds that female caretakers communicated significantly more with female adolescents (mean; Female adolescents = 7.95 vs. Male adolescents = 7.62) and male caretakers communicated significantly more with male adolescents (mean, Male adolescents = 8.13 vs. Female adolescents = 7.96). Moreover, both male and female caretakers who are staying with their biological adolescents communicated significantly more frequent with many topics of SRH to their adolescents than their counterparts. Female caretakers with higher education level and those of age group of 50 to 59 years were more likely to communicate with their adolescents, however there was no significant difference in communication among male caretakers with different education level and within different age groups (Table 4).

**Table 4:** Means of overall communication among caretakers of adolescents (*N* = 1000)

Characteristics	COMMUNICATION MEAN SCORE			
	Female (n = 795)		Male (n = 205)	
	Mean (SD)	*p*-value ^a^	Mean (SD)	*p- value* ^a^
Age groups (years)				
35–39	9.13 (4.1)	<0.001	10.52 (4.7)	0.66
40–49	10.68 (4.9)		11.23 (5.7)	
50–59	11.61 (4.9)		11.08 (4.8)	
60+	10.26 (4.8)		10.15 (4.6)	
Level of education				
No formal education	9.38 (4.6)	0.009	11.55 (5.4)	0.25
Primary education	10.05 (4.6)		10.76 (5.4)	
Secondary and Higher education	10.69 (4.7)		10.12 (4.1)	
Relationship with adolescent				
Biological adolescent	9.55 (4.4)	<0.001	9.78 (4.3)	<0.001
Non biological adolescent	7.65 (2.3)		7.04 (2.4)	
Have both biological and non biological adolescent	13.12 (5.0)		14.98 (4.9)	
Caretaker’s adolescents’ sex				
Caretakers having male adolescents only	7.62 (2.5)	<0.001	8.13 (2.4)	<0.001
Caretakers having female adolescents only	7.95 (2.2)		7.96 (2.6)	
Caretakers having both male and female adolescents	15.40 (4.4)		15.45 (4.5)	

^a^Results represent an ANOVA test

When comparing with female, male caretakers communicated significantly more frequent to female adolescents about pregnancy, safer sex, abortion and homosexuality, however; there was no significant difference in communicating different SRH topics to male adolescents among male and female caretakers. The assessment of SRH topics that are mostly discussed by caretakers revealed that abstaining from sexual activities was the most frequently discussed topic to female adolescents by both male 56 (68.3%) and female 209 (63%) caretakers, and it is the least discussed topic to male adolescents by female caretakers 85 (39.2%). HIV/STIs was the most discussed topic to male adolescents by female 103 (47.5%) and pregnancy was most discussed by male caretakers 29 (59.2%), while safer sex (M = 47.6% vs. F = 38.3%) and contraceptive use (M = 39.0% vs. F = 43.7%) were the least discussed topics to female adolescents by both male and female caretakers (Table 5).

**Table 5:** Sex difference in reporting frequency of communication on specific topic of SRH by sex of adolescents (*N* = 1000)

	COMMUNICATION AMONG CARETAKERS (sometimes and a lot)			
SRH topic and sex of adolescent	Male	Female	*χ* ^2^	*p - value*
	Number (%)	Number (%)		
HIV and STIs				
Male adolescents	27 (55.1)	103 (47.5)	3.2	0.36
Female adolescents	40 (48.8)	142 (55.0)	7.1	0.06
Pregnancy				
Male adolescents	29 (59.2)	93 (42.9)	6.7	0.08
Female adolescents	43 (52.4)	140 (42.1)	15.39	0.02
Abortion				
Male adolescents	25 (51.0)	90 (41.5)	1.6	0.45
Female adolescents	45 (54.9)	135 (40.7)	12.4	0.002
Abstaining from sex				
Male adolescents	25 (51.0)	85 (39.2)	3.50	0.32
Female adolescents	56 (68.3)	209 (63)	1.99	0.57
Contraceptive				
Male adolescents	23 (46.9)	90 (41.8)	1.05	0.79
Female adolescents	32 (39.0)	145 (43.7)	2.31	0.51
Safer sex				
Male adolescents	22 (44.9)	88 (40.5)	0.9	0.83
Female adolescents	39 (47.6)	127 (38.3)	11.61	0.009
Homosexuality				
Male adolescents	26 (53.1)	91 (42.0)	4.2	0.23
Female adolescents	44 (53.7)	140 (42.2)	9.01	0.03

### Correlation among IMB constructs and communication practice

The overall score, mean and standard deviation of information, motivation and behavioral skills constructs are summarized in Table 6. There was no significant difference between male and female in reporting the elements of IMB constructs except for social norms in which male caretakers reported to have significantly less restrictive social norms (7.89 vs. 7.63) on communicating SRH matters with adolescents compared to female (Table 7).

**Table 6:** Mean score for IMB constructs and communication variables (*N* = 1000)

Variable		Total points	Range (Min-Max)	Mean ± SD
I	Information	15	4–15	10 ± 1.96
M	Perceived risk	12	2–10	5.4 ± 1.58
	Social norms	12	4–12	7.6 ± 1.41
	Attitude	20	8–20	13.3 ± 9.10
B	Perceived self efficacy	16	4–16	8.4 ± 2.40
	Perceived objective skills	16	4–14	7.4 ± 2.50
Outcome	Communication practice	36	9–30	18.8 ± 5.10

**Table 7:** Sex differences in reporting IMB constructs (*N* = 1000)

Characteristics	Male		Female		*t*	*p – value*
	Mean	SD	Mean	SD		
INFORMATION	10.05	2.21	10.09	1.89	0.26	0.79
MOTIVATION						
Perceive more risk	5.38	1.62	5.43	1.58	0.41	0.68
Have less restrictive social norms	7.89	1.29	7.63	1.44	−2.42	0.01
Positive attitude	13.15	1.64	13.38	2.00	1.57	0.11
BEHAVIORAL SKILLS						
Perceive have high self efficacy	8.52	2,57	8.36	2.42	−0.79	0.42
Perceive have adequate skills	7.60	2.54	7.42	2.58	−0.89	0.37

Communication was found to correlate with perceiving high risk, restrictive social norms, perceiving high self efficacy and perceiving effective skills. Overall, it is suggested that IMB constructs were inter-related and associated with communication behaviour (Table 8). These pattern of association supporting moving forward with test of the IMB model pertaining to communication practice.

**Table 8:** Correlation coefficients for IMB constructs and communication (*N* = 1000)

	Communication	Perceived Skills	Perceived Self efficacy	Attitude	Social Norms	Perceived Risk
Knowledge	0.015	0.034	0.007	−0.055	0.028	−0.076*
Perceived risk	0.072*	0.085**	−0.024	0.003	−0.158^**^	
Social norms	−0.092^**^	−0.149**	−0.108^**^	−0.036		
Attitude	0.034	−0.035	−0.130**			
Perceived Self efficacy	0.222**	0.384**				
Perceived Skills	0.240**					

*p < 0.05

**p < 0.001

### Test of IMB model

A simple path analysis via regression was conducted. For the IMB model, two “layers” of multiple regressions were run: the first one with behavioral skills as the criterion and information and indicators of motivation as the predictors. The second one with communication practice as the criterion and information, indicators of motivation and indicators behavioral skills as the predictors. The results show that, indicators of behavioral skills influences communication practice. Motivation through its element of attitude shows direct effect to communication while the element of perceived risk and social norms of motivation construct has indirect effect to communication through behavioral skills. Information on the other hand has neither direct nor indirect effect to communication (Table 9).

**Table 9:** Regression coefficients for Communication and behavioral skills (*N* = 1000)

Communication practice				Behavioral skills		
	*B*	Beta	*t*	*B*	Beta	*t*
(Constant)	3.016		1.638	21.571		14.413
Information	0.042	0.017	0.561	0.054	0.025	0.810
Perceived risks	0.170	0.057	1.828	0.042	0.016	0.507
social norms	−0.132	−0.039	−1.256	−0.464	−0.157**	−4.966
Attitude	0.148	0.060*	1.957	−0.220	−0.102**	−3.269
Perceived efficacy	0.314	0.162**	4.861			
Perceived skills	0.313	0.169**	5.084			

*p < 0.05

**p < 0.001

## **Discussion**

In this study we assessed the pattern of caretaker-adolescent communication on SRH and the sexual topics that caretakers discuss to either male or female adolescents. IMB model was used as a framework in which Information (knowledge on SRH), Motivation (perceived risk, social norms and attitude), and Behavioral skills (perceived self efficacy and perceived objective skills in communicating SRH matters with adolescents) were assessed and their pattern of association to communication practice was reported. The findings of the present study represent one of the only quantitative reports which describes the pattern of SRH communication as described by caretakers of adolescents in Tanzania, as many study of parent-child communication relied on the information as reported by adolescents [12–17].

As pointed out in the result, a low proportion of caretakers in Unguja-Zanzibar communicate with their adolescent about SRH issues. The findings show that only 40.7% of caretakers had ever communicated with their adolescents on SRH issues and a very low percentage of 9.2 caretakers reported to have had communicated in the past 30 days. On the other hand, assessment of communication using weighted-specific topics measure revealed that only 26.5% of caretakers reported to have had communicated more frequently with many topics of SRH with their adolescents. Although both communication measures yielded a low communication prevalence, the topic-specific communication measure is more effective and more preferred compared to global form because the global measures may fail to capture the all aspects of sexual communication and may often result in interpretation bias [21], while topic-specific communication measures both the number of topics discussed and the extent of that discussion.

The low communication prevalence found in this study is not a surprising finding in the context of Zanzibaris cultural background where traditionally, sexual communication is perceived to be a taboo and likely to encourage premarital sexual activities [18]. This probably concur the concept that sexual communication in the African context is rare [28]. However, recent studies in some African countries have found higher levels of parent–child communication [20, 29, 30] the situation is seem to be different in Tanzania. The difference was also observed in earlier studies which found a moderate amount of SRH communication in some African countries but was low in Tanzania. For example a study of Namisi et al., (2009) reported that non-communication was more common in Dar-es Salaam varied between 69.3% and 86.6% than in two South African sites in which the proportions were between 25.5% and 60.3% [12]. Although the above findings were from the perspective adolescents, it can be counted as an indication of communication prevalence in Tanzania. The current study findings therefore affirmed the trends in Tanzania.

The communication pattern varies across gender of caretaker and that of adolescent and also depends on the nature of relationship between caretaker and adolescent [31–33]. The findings of this study show that same sex communication was more common compared with the communication to the opposite sex. In his study Izugbara (2008), reported similar findings that female parents discussed sex related issues only with their female children, and male parents discussed only with their male children [34]. Same sex discussion is preferred because of feeling of shame and embarrassment when discussing SRH matters with the opposite sex [35]. Though it is preferred by most caretakers and adolescents [12, 31], same sex discussion is not sensible to promote because there may be a time when caretakers are obliged to exercise both roles as a mothers and fathers to their male or female adolescents, example in a situation when one spouse dies, or get divorced or having irresponsible spouse. In these cases, the adolescent of the opposite sex to that of caretaker may be deprived of SRH information. Therefore there is a need to encourage SRH discussion to both sex of adolescents by providing required communication skills and motivation to caretakers as well as to adolescents.

Caretakers found to communicate more with their female adolescents rather than male adolescents. One study in Nigeria explored that young women are generally viewed as very prone to deceptions and likely to make mistakes that could ruin their future, that is why parents believed that discussing matters of SRH with female children was more important than doing so with male children [34]. However it is not clear to us why caretakers in Unguja like to discuss SRH matters more with female adolescents. Although these gender differences exist in parent-adolescent communication, both parents may influence adolescents’ sexual risk-taking behaviours [35]. Therefore further researches are needed to explore this area.

Communication about abstinence from sex appeared as the common topic discussed to female adolescents by both female and male caretakers. On the other hand, HIV/STIs was mostly discussed to male adolescents by female caretakers and pregnancy by male caretakers. The rarely discussed topics to female adolescents by both sexes of caretakers are condom and other contraceptive use while to male adolescents, abstinence is rarely discussed by female caretaker and safer sex by male adolescents. Similar topics reported in many studies as the focus of discussion and the least discussed topics between caretakers and adolescents [5, 23, 34, 36], however studies did not differentiate topics communicated by different sex of caretaker to different sex of adolescents. This is the only study which report in details different sex-choice of topic of discussion by gender of adolescents. Further researches are needed to explore the reason behind the gender differences in SRH topics selection.

### Evaluation of IMB model in relation to communication practice

Results show that caretakers who communicate SRH matters with their adolescents perceived themselves as having high self efficacy, effective objective skills and having positive attitude. These bivariate correlation findings support previous researches [37, 38] which showed that parents perception of their ability to speak with their child about sexual issues (HIV, condom use) has been associated with parent–adolescent communication. As hypothesized by the IMB model, this study finds that behavioral skills is an important predictor of communication. Despite the significance of behavioral skill in the development of communication behavior, many studies revealed that parent lack these skills and it could be one among the major reason of little or no parent-child discussion observed especially in Sub Saharan Africa countries [28].

The indicators of motivation in this study show inverse relation with behavioral skills, which is contrary to the IMB model stipulation. In fact those who have less restrictive social norms and positive attitude have low skills and efficacy and communicate less. Similar observation was found in the study [39] in which motivational indicators were correlated in opposite directions with risk behavior. This inverse relation could partly be explained by the response biased that could be caused by research interest desirability and or potential embarrassment that may have lead to concealment of behaviors and therefore under or over-reporting. While it is possible that the results do, in fact, accurately reflect a negative influence of perceived risk, social norms and attitude on behavioral skills and communication behaviour for our sample, we are limited in our conclusions by the nature of the measurements we used. It is possible that our measures for the motivation construct lacked sufficient sensitivity due to limited item sets. However, it is encouraging to observe the significant relationship exist between motivation and communication practice. Further researches are needed to explore the role of motivation in communication practice.

Information construct on the other hand has a negligible influence on communication. In fact, we observed a considerable variability in communication behavior despite the high score of knowledge on SRH, and therefore we conclude that SRH knowledge alone is insufficient to influence the communication behaviour. This is not surprising, given previous research showing that information is often insufficient to change the behaviour [40, 41] Other researchers call into question on the role of information or knowledge in behavior change in the context of the IMB model [39]. However, according to Mezzuca [42], the IMB model holds that information is a prerequisite for changing behavior, but in itself is insufficient to achieve this change. Information about SRH in this study may have been unrelated to communication behavior may be due to the type of information that was assessed, or because information about SRH is really not as influential of its practice compared behavioral skills. Additional studies are needed to identify whether the information construct for communication or its measurement are problematic.

The current study finding are likely to have important theoretical and practical implications for refining parent-child SRH communication interventions. However, these implications are constrained by methodological limitations of the current research. Our study, relied on self-report behavioural instruments in which caretakers were asked retrospectively on their communication practice to their adolescents on mentioned topics of SRH without counterchecking their response on the side of adolescents. The quality of such communication was also not determined. Social desirability and recall bias can create response biases and may have lead to over or under -reporting communication behaviours.

## Conclusion

In conclusion, our findings suggest that caretakers-adolescents communication on SRH in Unguja is low and when it occurs, it is more prevalent among caretakers having female adolescents, having biological relationship with adolescent, and between same sex. Sexual abstinence is mostly discussed to female adolescents by both sex of caretakers, while pregnancy and HIV/STIs are mostly discussed to male by male and female caretakers respectively. Safer sex and contraceptives use were rarely discussed to both sex of adolescents by both sex of caretakers. From this study findings, it is indicated that some communication takes place on particular reproductive health issues but not others. In particular caretakers fail to communicate with their adolescents children on sensitive issues of sexuality like condom use, homosexuality and abortion, but do so on less sensitive ones such as abstinence and HIV. This implies that communication between caretakers and adolescents is not comprehensive and informative when it comes to preparing their growing children to handle the emerging sexual needs they feel responsibly, so that they can minimize the risks for early pregnancy, STIs and HIV infection. Interventions programmes ought to include strategies that enhance caretaker’s information, motivation, skills and confidence in communicating SRH matters so as to improve caretaker-adolescent communication. The current findings are likely to offer new information regarding the use of the IMB model in designing intervention to improve parent-child communication behaviours.

## Abbreviations

HIV: Human Immunodeficiency Virus
IMB: Information Motivation Behavioral skills model
SRH: Sexual and Reproductive Health
STIs: Sexual Transmitted Infections
